# Human renal angiomyolipoma cells of male and female origin can migrate and are influenced by microenvironmental factors

**DOI:** 10.1371/journal.pone.0199371

**Published:** 2018-06-19

**Authors:** Francesca Bertolini, Giulia Casarotti, Luisella Righi, Enrico Bollito, Carlo Albera, Silvia Anna Racca, Donato Colangelo, Barbara Mognetti

**Affiliations:** 1 Department of Clinical and Biological Science, University of Turin, Orbassano, Italy; 2 Department of Health Sciences, Università del Piemonte Orientale, Novara, Italy; 3 Pathology Unit, Department of Oncology, University of Turin, Orbassano, Italy; 4 Pathology Unit, San Luigi Gonzaga Hospital, Orbassano, Torino, Italy; Duke University School of Medicine, UNITED STATES

## Abstract

**Background:**

Improving the knowledge of angiomyolipoma physiopathology might help in refining its pharmacological treatment. We investigated if angiomyolipoma cells have migratory properties, how their growth and motility can be influenced by the hormonal milieu, and if this can be related to a specific gender.

**Methods:**

Primary cells were isolated from angiomyolipomas surgically resected for therapeutical reasons in a female and in a male patient. The genetic control demonstrated no TSC2 deletion. Bi- (wound healing) and three-dimensional (transwell assay) migration were analyzed in vitro in basal conditions and under the influence of 17- β-estradiol and SDF-1α.

**Results:**

Treatment up to 72 hours with 17-β-estradiol (0.1–100 nM), tamoxifen (0.2–20 μM) or with both, does not modify angiomyolipoma cells proliferation. On the other hand, SDF-1α and 17-β-estradiol treatment induce a significant motility increase (both bi- and three-dimensional) which becomes evident already after 2 hours of incubation. Angiomyolipoma cells express mRNA coding for SDF-1α and 17-β-estradiol receptors and secrete both the metalloproteases principally involved in malignant phenotype acquisition, i.e. MMP-2 and MMP-9.

**Conclusion:**

Angiomyolipoma cells behave similarly, despite their different source. Primary angiomyolipoma cells migrate in response to hormonal milieu and soluble factors, and produce active metalloproteases, both aspects being consistent with the theory claiming they can migrate to the lungs (and/or other organs) and colonizing them. No main feature, among the aspects we analyzed, seems to be referable to the gender of origin.

## Introduction

Widespread use of cross-sectional imaging of kidneys has resulted in a significant increase in incidentally diagnosed small masses. The prognosis is usually favourable since they rarely progress to metastases [1]. Angiomyolipomas (AMLs) most commonly occur in the kidneys as small masses and, although often asymptomatic, may enlarge and bleed leading to haemorrhage and renal impairment [2]. These mesenchymal lesions are characterized by proliferation of spindle cells, epithelioid cells and adipocytic cells in concert with many thick-walled blood vessels [3]. A normal tissue counterpart has not been identified and genetic analyses indicate that all three tissue components derive from a common progenitor cell [4,5]. In case of intractable pain, large mass size (>4 cm) and risk of bleeding, surgical intervention is needed [6]. The preferred treatment for AML is nephron-sparing surgery or selective renal artery embolization, since both methods preserve residual renal function in comparison to radical nephrectomy [7]. On the other hand, asymptomatic patients are managed conservatively with long-term surveillance (mainly by imaging).

Although most AMLs are clinically insignificant benign tumors, an uncommon subtype, the epithelioid AML, can behave more aggressively and develop distant metastases [8,9,10].

AMLs are twice as common in females, and can occur sporadically or in association with other disorders, such the autosomal dominant condition Tuberous Sclerosis Complex (TSC) and sporadic lymphangioleiomyomatosis (LAM). In particular, LAM is a progressive disease of the lung histologically characterized by a diffuse proliferation of atypical smooth muscle cells (LAM cells) in the alveoli and cystic degeneration of the normal lung parenchyma [11].

AML and LAM share the same origin from mesenchymal perivascular epithelioid cell (PEC) and therefore both are considered as belonging to the PEComas lesion family [12]. The smooth muscle–like LAM cells that diffusely infiltrate the lungs and the lymphatic vessels have a low proliferation index and little or no evidence of cellular atypia. In the handful of patients who have had multiple tissues available for sequencing, identical inactivating mutations of TSC1 or TSC2, with subsequent deregulation of the Rheb/mTOR/p70S6K pathway, were demonstrated in AML, in lymph nodes, and in pulmonary LAM cells, but not in normal lung of the same patient [13].

It has been also shown that both AML and LAM cells share immune-expression of HMB-45 antigen [14,15].

Furthermore, both LAM and AML cells express estrogen receptor α [16], and estrogen is thought to cause clinical worsening in women with LAM [17]. Even more strikingly than AML, LAM preferentially affects women, especially at childbearing age, more often than AML. To date the underlying reasons for this behaviour are not known.

These, and other data [18,19], support a model in which both LAM and AML pathogenesis share some genetic and biological mechanisms, and are consistent with the hypothesis that pulmonary LAM might result from the metastatic spread of AML smooth muscle cells [20], possibly influenced by the hormonal milieu.

Therefore, considering the diffuse approach of delaying AML ablation in asymptomatic patients to preserve renal function, and that there is no reliable imaging technique able to differentiate a benign AML from one undergoing malignant change, we deem of paramount importance the study of migratory properties of AML cells. A clear demonstration that AML cells migration is involved in pathogenesis of lesions in several different organs might suggest important hints on new preventive drug therapies.

For this reason, we undertook a study on the proliferative and migratory properties of primary cells isolated from two different surgically removed AMLs, respectively from a male and a female patient. We evaluated if 17-β-estradiol could modulate their growth and their two- and three-dimensional migration. Furthermore, we compared the hormone-dependent effects to those induced by the stromal cell-derived factor 1 α (SDF-1α), a soluble factor known to induce cellular migration [21]. We also investigated metalloproteases 2 and 9 (MMP-2 and MMP-9) activities of AML cells, both in basic and stimulated conditions, since these MMPs play a pivotal role in the pathogenesis of cystic lung destruction in LAM [22,23].

MMPs modification of the extracellular matrix usually contributes to cell migration as well as to tissue invasion and metastasis. Similar modifications may facilitate AML cell migration and pulmonary colonization [20]. In fact, MMPs imbalance, together with other factors like a strong expression of cathepsin K, bcl-2 and HMB-45, characterize this pathology [19].

We focused our attention on estrogens in order to investigate if there are some differences in AML cells behaviour according to the hormonal milieu, as suggested by the hypothesis that pulmonary LAM in women might derive from the metastatic spread of AML abnormal smooth muscle cells.

## Materials and methods

### Materials

All reagents were purchased from Sigma (St. Louis, MO, USA) unless otherwise stated. Tissue culture plasticware was from Falcon (Franklin Lakes, NJ, USA).

### Angiomyolipoma cells, tissue and ethical approvals

Human primary AML cells have been obtained from patients that underwent surgical nephron-sparing AML ablation for therapeutic purposes at the Urology Unit of the San Luigi Gonzaga Hospital. The study was approved by the Ethical Committee of the San Luigi Gonzaga Hospital, University of Turin, Orbassano, Turin, Italy (Protocol 0006771, approved on April 18, 2016). All patients provided written informed consent in accordance with the Declaration of Helsinki.

AML3 derives from a male patient, AML4 from a female patient. None of the patients had any clinical signs or symptoms or a family history of tuberous sclerosis. No genetic analysis of TSC mutation were performed.

AML diagnoses were confirmed by standard histological examination including specific immunostaining for alpha-smooth muscle Actin, HMB-45 and Pancytokeratin antigens.

Primary cells were isolated from excess material not required for diagnostic use, which was divided into small fragments and treated with type II collagenase. Resulting cell suspensions were plated into T25 tissue culture flasks in AML medium (adapted from Lesma et al. [24]), composed of phenol red DMEM medium, ferrous sulphate 1.6 μM, 20% foetal calf serum (FCS) and 10 μg/mL epidermal growth factor. Experiments were performed on cells at passage 3–6.

### DNA sequencing

Total genomic DNA was purified from primary cells using Maxwell^®^16 Cell LEV DNA Purification Kit (Promega, Italy), according to the manufacturer’s instructions.

Direct sequencing by Sanger method of TSC2 exons 1–42 and exon-intron splicing junction boundaries was performed as routinely by the Laboratory of Molecular Genetics.

PCR reactions were treated with exonuclease I and shrimp alkaline phosphatase (ExoSAP-IT, USP Corporation, Ohio, USA) and sequence reactions were performed by ABI BigDye® Terminator kit (Applied Biosystems, Foster City, CA, USA) and analysed by ABI PRISM 3130xl Genetic Analyzer (Applied Biosystems, Foster City, CA, USA).

Sequence data were analyzed using Mutation surveyor DNA variant analysis software (Softgenetic PA, USA).

### Immunofluorescence

AML cells were fixed in 4% paraformaldehyde (PAF) for 15 minutes. After washing in PBS cells were treated with PBS containing 1% normal goat serum (NGS), 0.1% Triton X-100 at room temperature (RT) for 1 hour. Cells were incubated overnight at 4°C with the following primary antibodies against: S-100 (rabbit, 1:800; Dako, Glostrup, Denmark), α-Smooth-Muscle Actin (mouse, 1:100; NeoMarkers, Fremont, CA), HMB45 (mouse,1:100, Dako, Glostrup, Denmark), Keratin 8/18 (mouse, 1:100 Menarini, Florence, Italy) Vimentin (mouse, 1:70; Novocastra Lab, Newcastle, UK) and Melan-a (mouse; 1:100; NeoMarkers, Fremont, CA). After washing, cells were incubated for 1 hour at RT with the appropriate secondary antibodies: goat anti-mouse IgG Alexa-Fluor-488-conjugated (1:200, Molecular Probes, Eugene, Oregon) and CY3-conjugated anti-rabbit IgG (dilution 1: 400, Dako, Milan, Italy) [25]. The immunostained coverslips were analyzed on a Zeiss fluorescence microscope and images were captured with an Axiovision Imaging System.

### 2D migration assay-wound healing

Two-dimensional migration assays were performed as described in Mognetti et al. [26]. Briefly, primary AML cells were seeded in a 12-well plate at 300,000 cells/well. When they were confluent, a cross “wound” was made in each well with a p1000 tip [27], then wells were washed thrice with PBS, and cultured in AML medium supplemented either with SDF-1α (Peprotech, London, UK) 100 ng/mL, plerixafor 100 nM, 17-β-estradiol 1 nM, or tamoxifen 2 μM. We photographed “wounds” on time-laps every hour to highlight migration, until a maximum of 8 hours.

Experiments were repeated three times, and every time five different spots for each experimental condition were considered. Images were analysed using ImageJ software (Wayne Rasband, NIH, USA): the healing percentage was quantified comparing the wound area at t = 0 to the following time-points for each treatment.

### 3D migration assay

The transwell migration assay, performed as previously described in Mognetti et al. [28], was used to measure the three-dimensional movements of cells. Migration assays were performed in transwells (BD Falcon cell culture inserts incorporating polyethylene terephthalate membrane with 8.0 μM pores, 6±2x10^4^ pores /cm^2^) in 24-well plates.

When tests were performed in the presence of SDF-1α blocker, cells were preincubated for 30 min at 37°C in 100 nM plerixafor conditioned medium.

Cells (5x10^4^) were suspended in 200 μL of culture medium and seeded in the upper chamber of a transwell. The lower chamber was filled with fresh culture medium with or without 100 ng/mL SDF-1α or 17-β-estradiol 1 nM, and placed in the incubator. After 4 or 8 hours, cells were treated as detailed by Gambarotta et al. [27]. Wells were photographed using a BRESSER MikroCam 3 Mpx camera, with an optical microscope (Leica DC 100) at 100x. Five pictures were randomly chosen *per* well and used to count the migrated cells with ImageJ software using cell-counter plug-in. Results from different experiments (performed at least three times in duplicate) were expressed as mean ± standard errors. In order to avoid any cytotoxic effect potentially confounding migration results, we performed a cytotoxicity test at the same time and same conditions of every migration test.

### Proliferation assay

Primary AML cells were seeded into flat-bottomed 96-well microplates at a density of 1,000 cells in 100 μL culture medium *per* well and allowed to attach overnight in complete medium. Drugs were then added to culture medium at concentrations ranging from 0.1 to 100 nM 17-β-estradiol, and from 0.2 to 20 μM tamoxifen [29] for 24–72 hours, according to protocols. The MTT (3-(4,5-dimethylthiazol-2-yl)-2,5-diphenyltetrazolium bromide) assay was performed as described in Mognetti et al. [28]. Data (mean ± standard errors) were calculated as the mean values of 8 replicates. Each experiment was repeated thrice. Cell viability was expressed as the percentage of living cells *versus* the untreated controls.

### Quantitative real-time PCR (qPCR)

In order to perform quantitative real-time PCR (qPCR), total RNA was extracted from treated cells by using Trizol (Invitrogen Life Technologies, Italy). After RNA purification and treatment with DNAse I (Fermentas, St. Leon-Rot, Germany), 1 μg was retrotranscribed in cDNA with the RevertAid™ H Minus First Strand cDNA Synthesis Kit (Fermentas) using oligo(dT) primers. Gene assays were performed in triplicate for each treatment in a 20 μL reaction volume containing 1 μL of RT products, 10 μL Sso-Fast EVA Green SMX (Bio-Rad, Hercules, CA, USA), 500 nM each forward and reverse primers. Gene expression was normalized on the housekeeping gene ribosomal 18S rRNA. Table 1 resumes the primer sequences that were used. Automated CFX96 real-time thermocycler (Bio-Rad) was used and the reaction conditions were 95°C for 1 minute, followed by 45 cycles 98°C for 5 seconds and anneal–extend step for 5 seconds at 60°C, with data collection. At the end of these cycles, a melting curve (65°C to 95°C, with plate read every 0.5°C) was performed in order to assess the specificity of the amplification product by single peak melting temperature verification. Results were analysed with Bio-Rad CFX Manager. Calculations and statistical analyses were performed using GraphPad Prism version 5.00 for Windows (GraphPad Software, San Diego California, USA).

**Table 1:** Primers sequences, size of the amplification product and NCBI reference sequence.

GENE	SEQUENCE		AMPL. SIZE	NCBI REF. SEQ.
**Erα**	Fw:	5’-TGGAGTCTGGTCCTGTGAGG-3’	172 bp	NT_025741.16
	Rev:	5’-CCCACCTTTCATCATTCCCACT-3’		
**Erβ**	Fw:	5’-GAGCAAAGATGAGCTTGCCG-3’	142 bp	NM_001437.2
	Rev:	5’-AGCTGGGCCAAGAAGATTCC-3’		
**GPR30**	Fw:	5’-AGTCGGATGTGAGGTTCAG-3’	240 bp	NM_001505.2
	Rev:	5’-TCTGTGTGAGGAGTGCAAG-3’		
**MMP-2**	Fw:	5’-GGCCCTGTCACTCCTGAGAT-3’	474 bp	NM_001302510.1
	Rev:	5’-GGCATCCAGGTTATCGGGGA-3’		
**MMP-9**	Fw:	5’-CAACATCACCTATTGGATCC-3’	480 bp	NM_004994.2
	Rev:	5’-CGGGTGTAGAGTCTCTCGCT-3’		
**18S rRNA**	Fw:	5’-GTGGAGCGATTTGTCTGGTT-3’	201 bp	X03205.1
	Rev:	5’-ACGCTGAGCCAGTCAGTGTA-3’		

Fw = forward, Rev = reverse

### Western blotting

Cells were seeded in 10 cm diameter Petri dishes, cultured until sub-confluence, then 17-β-estradiol (1 nM) was added. After 5 minutes and 4 hours incubation, cells were collected and treated as detailed by Mognetti et al. [21].

Blots were probed with primary monoclonal antibody anti-vinculin (mouse; 1: 2000 Sigma) resuspended in PBS Tween 0.1% and with anti-ERK1/2 (mouse; 1:2000), anti-pERK1/2 (mouse; 1:2000) (Cell Signaling Technology, Danvers, MA, USA) resuspended in 5% w/v nonfat dry milk + PBS tween 0.1%. Vinculin was used as an internal control. HRP-conjugated anti-mouse (Amersham-GE Healthcare, Buckinghamshire, UK) was diluted 1:6000 (ERK1/2 and pERK1/2) and 1:8000 (vinculin) in PBS Tween 0.1%. Densitometric analysis was performed by ImageJ software.

The ratio pERK1/2/ ERK1/2 was expressed as percentage optical density modification relative to control conditions. Experiments were repeated three times.

### Gelatin zymography

MMP-2 and MMP-9 activities in medium samples were assessed by gel zymography. Proteins (100 μg) were separated by electrophoresis in 8% SDS-PAGE gel containing gelatin (0.8 mg/mL) under non-reducing conditions. The gel was washed with Tris buffer (2.5% Triton X-100 in 50 mM Tris-HCl, pH 7.5, final solution) for 1 hour, then incubated overnight at 37°C in a proteolysis buffer (40 mM Tris-HCl, 200 mM NaCl, 10 mM CaCl_2_, 0.02% NaN_3_, pH 7.5, final solution). The gel was stained for 3 hours with Coomassie Blue solution (0.05% Coomassie Brilliant Blue R-250, 50% methanol, 10% acetic acid, final solution) and finally destained with 5% methanol and 7% acetic acid (final solution). Reagents and chemicals were obtained from VWR International (Milan, Italy). MMPs activity was detected as a clear band on a blue background and estimated by densitometric analysis using ImageJ Software. The results were expressed as percentages of control values.

### Statistical analysis

All the data in this study are shown as the mean ± SE. Two group means were compared using the unpaired t-test, and more than two group means were analyzed by one-way analysis of variance (ANOVA), where P <0.05 was considered statistically significant [30].

For gene expression level comparison One-way ANOVA with Dunnett’s post tests were performed using GraphPad Prism version 5.00 for Windows.

## Results

### Cell characterization by DNA sequencing and immunofluorescence

Direct sequencing by Sanger method of TSC2 exons 1–42 and exon-intron splicing junction boundaries of the TSC2 gene did not show any deleterious mutation in both samples.

**Fig 1:**
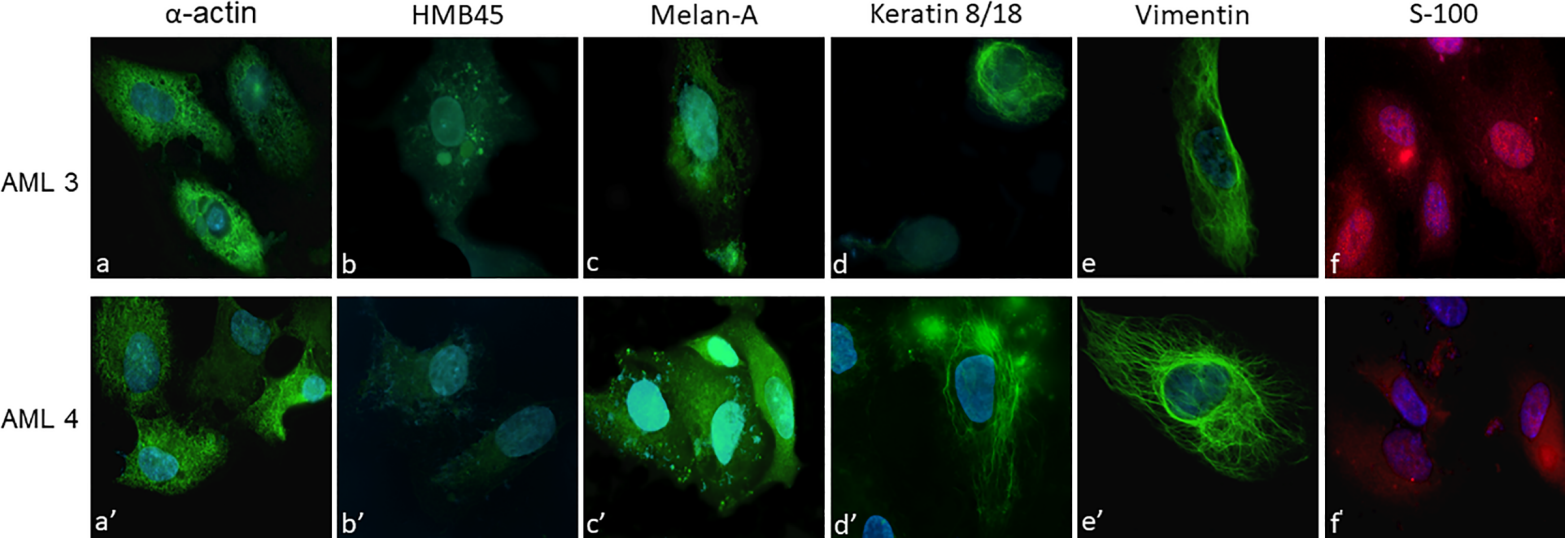
Primary angiomyolipoma cells characterization by immunofluorescence. Cells isolated from the two angiomyolipomas were challenged with specific antibodies to reveal their immunocytochemical characteristics: α-actin antibody (a and a’) specific for smooth muscle cells; HMB45 (b and b’) and Melan-A (c and c’), both typical of AML; keratin 8/18 (d and d’) labeling the epithelial-like cells; vimentin (e and e’), a marker of fibroblasts and S100 (f and f’), a marker of lipid-containing cells. Fields were chosen to show both the morphological aspect and the specific marker expression.

To better characterize isolated AML primary cells, immunofluorescence was performed with specific antibodies. Overall, both cell lines showed similar immunophenotype and partly elongated or rounded shapes. Both primary culture cells were strongly and totally positive for smooth muscle actin antibody (in both elongated and rounded cells), with a diffused stain throughout the cytoplasm (Fig 1A and 1A’). Furthermore, in both cell lines there were single rounded element positive for keratin 8/18 and for elongated cells strongly positive for vimentin (Fig 1D, 1E, 1D’ and 1E’), together with single negative ones for both the antigens. Finally a strong nuclear and cytoplasmic positivity was found for S100 in both cells (Fig 1F and 1F’). As a matter of fact, even if scattered, some AML3 cells were focally positive for intracytoplasmic HMB45 (Fig 1B and 1B’), and AML4 cells were focally positive for Melan-A antigen (Fig 1C and 1C’), which is consistent with the AML phenotype.

### Estrogen and SDF-1α receptors gene expression

**Fig 2:**
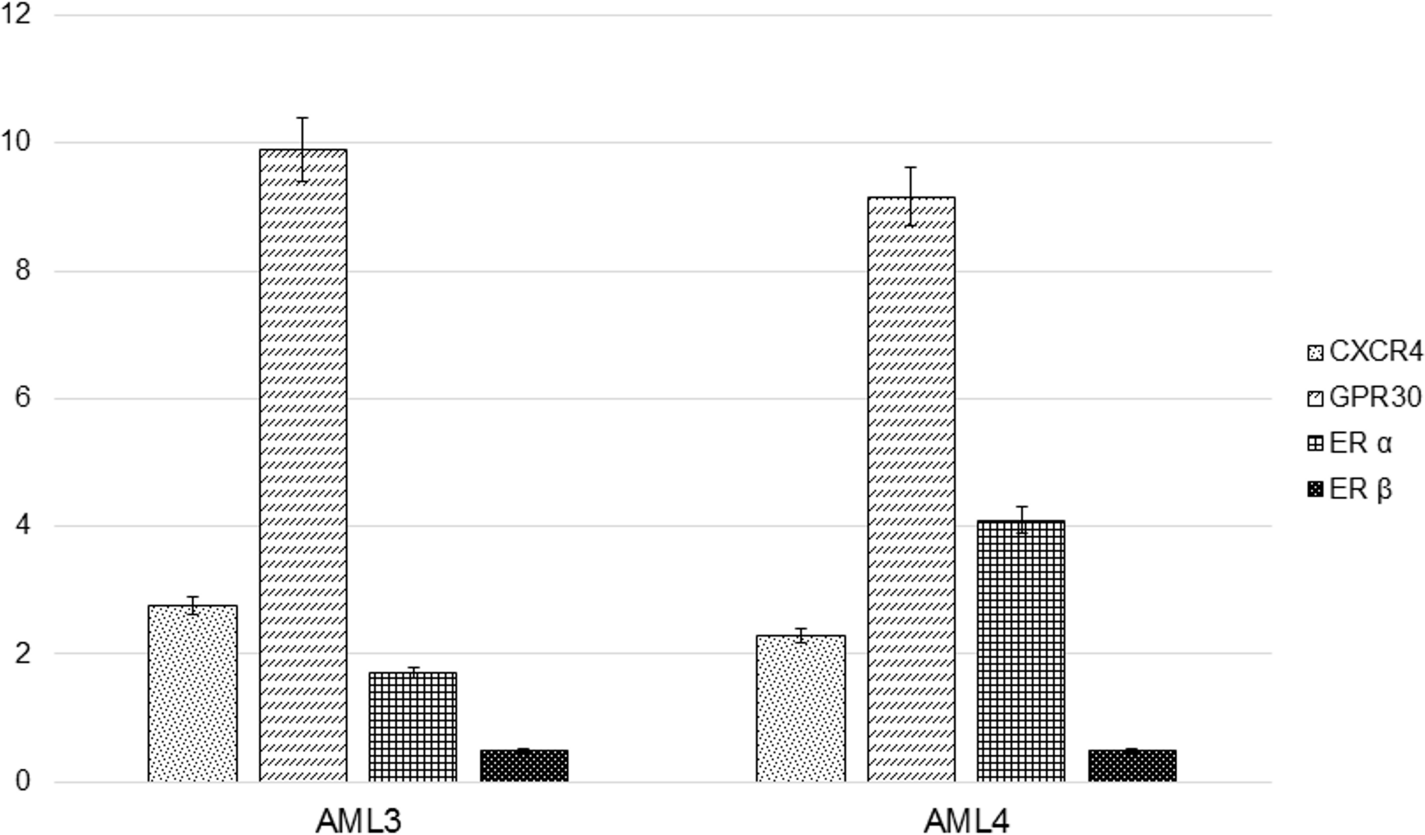
Receptors gene expression. Early passages AML cells underwent qRT-PCR for CXCR4, GPR30, ERα and ERβ mRNA expression analysis. Data are shown as the absolute mRNA expression normalized by the housekeeping 18S rRNA.

The analyses of gene expression of both AMLs demonstrated the presence of mRNA for CXCR4 (SDF-1α receptor), GPR30, ERα, but not for ERβ (Fig 2). The level of each gene was similar in both primary AML cells, with no significant difference.

### ERK phosphorylation

**Fig 3:**
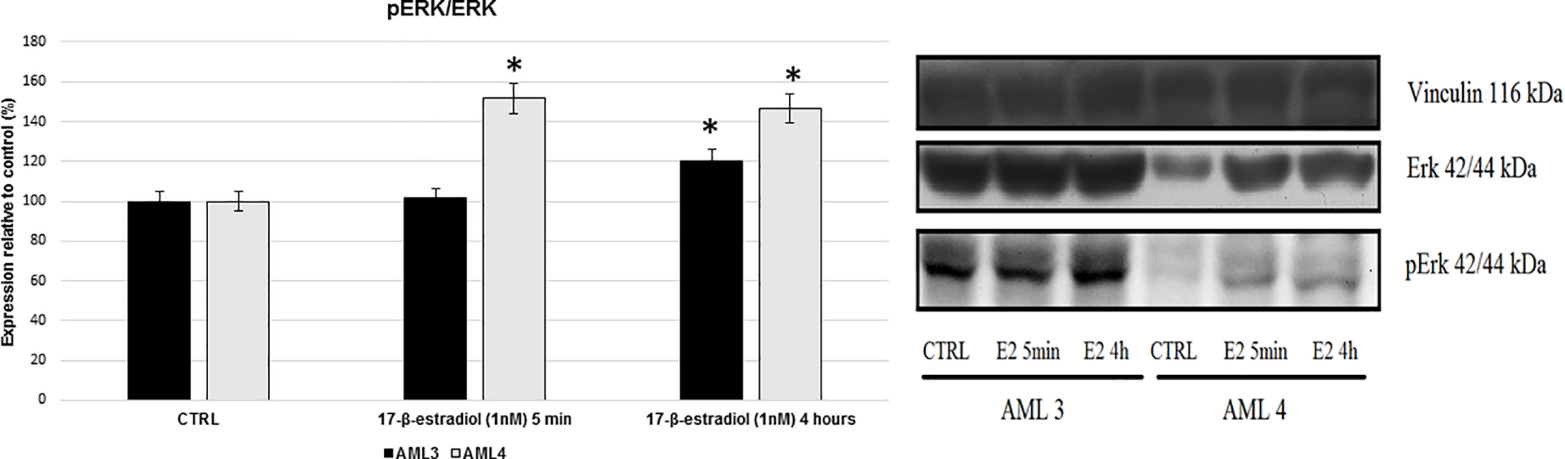
ERK phosphorylation. Effect of 17-β-estradiol (1 nM) on pERK/ERK in AML3 and AML4 cells after 5 minutes and 4 hours of incubation. Vinculin as internal control. * = P<0.05 vs control.

Treatment of AML3 cells with 17-β-estradiol increased ERK phosphorylation at 4 h, while pERK was significantly augmented in AML4 cells already at 5 minutes and was stable until 4 hours (Fig 3).

### Influence of 17-β-estradiol or tamoxifen on AML cell proliferation

**Fig 4:**
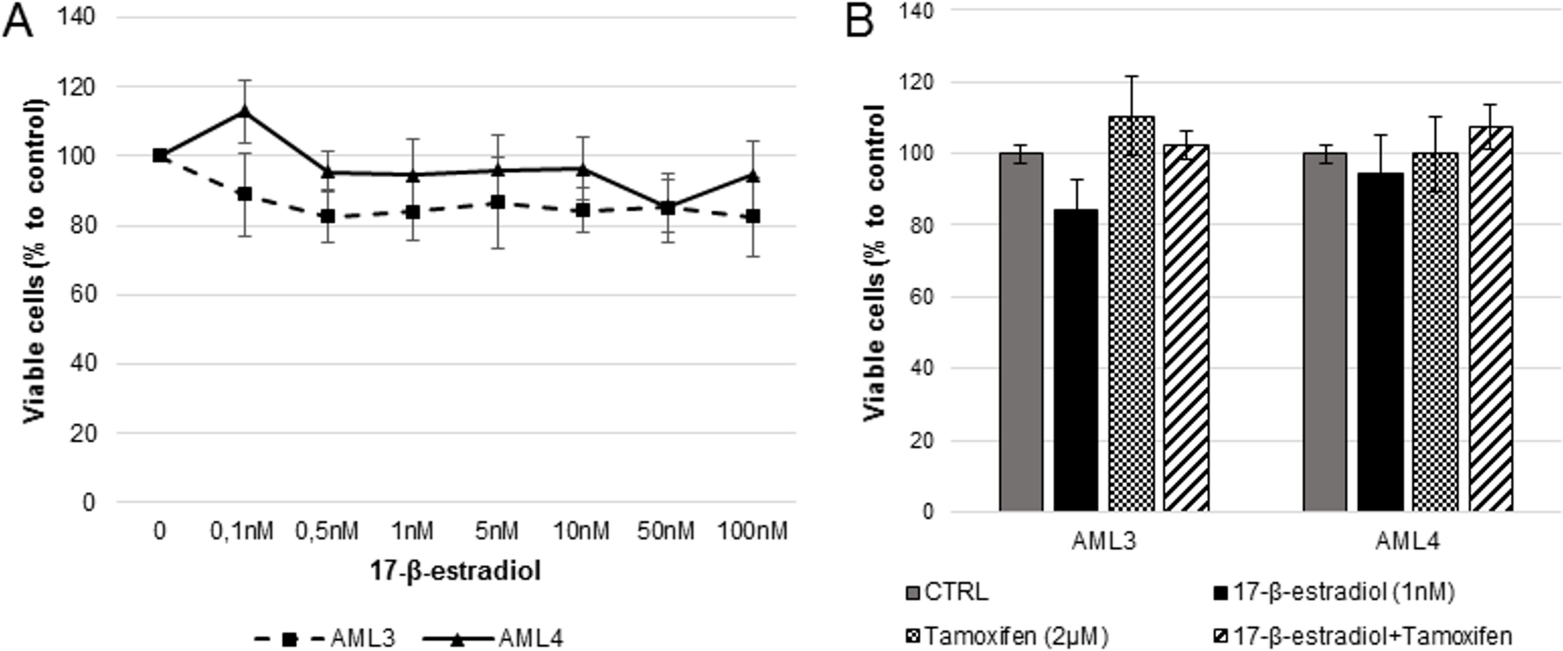
Effect of 17-β-estradiol alone and of its combination with tamoxifen on AML cells growth. Proliferation assay after 72 hours culture in presence of increasing concentration of 17-β-estradiol (A) or with 17-β-estradiol 1 nM, tamoxifen 2 μM, or both (B). Anova and Dunnett’s post test analyses demonstrated that no significant modification in cell growth respect to untreated controls was induced by the molecules at any of the concentrations tested.

The treatment for up to 72 hours with concentrations of 17-β-estradiol ranging from 0.1 nM to 100 nM with or without tamoxifen, or with tamoxifen alone (0.2–20 μM, two representative experiments are shown in Fig 4) did not induce any modification on AML cells proliferation regardless of their gender. No significant difference was detected at any time point or culture condition (data not shown). Fig 4 reports two typical experiments as an example.

### Two-dimensional motility assay (wound healing)

**Fig 5:**
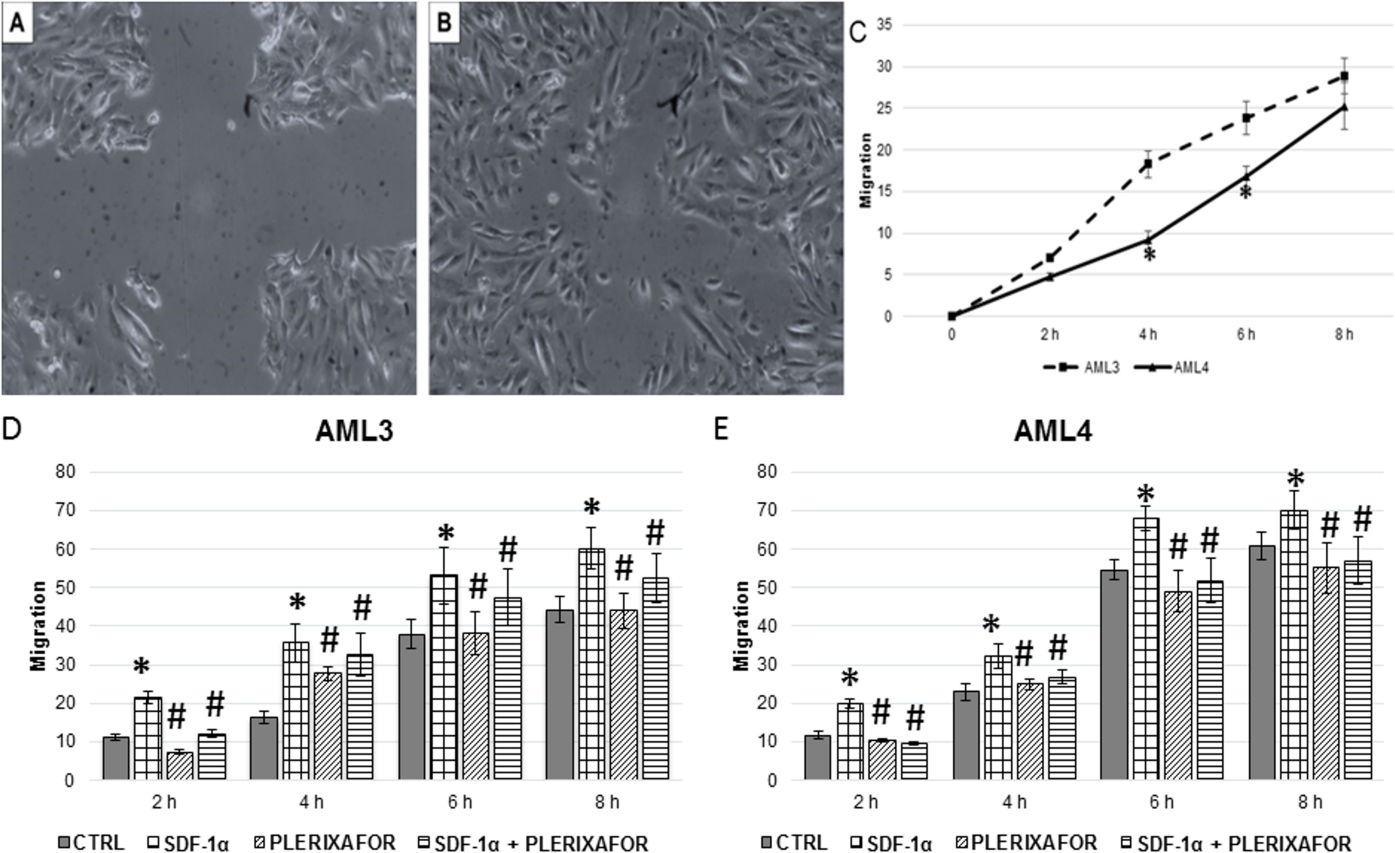
Analyses of primary AML cells migration by *in vitro* wound healing assay. Wounded area in a representative experiment of *in vitro* wound healing assay is shown before (A) and after (B) the incubation period. Bi-dimensional migration was then quantified in basal conditions for both AMLs (C), and for AML3 (D) and AML4 (E) in presence of SDF-1α 100 ng/mL or its receptor antagonist plerixafor 100 nM, or a combination of both. Migration is expressed in arbitrary units. * = P<0.05 vs control; ^#^ = P<0.05 vs SDF-1α.

The two-dimensional motility was quantified, and data are displayed graphically as healing percentage (Fig 5, panels C, D and E). Panel C compares basal migration: the early migration rate of AML3 was higher, a significant difference being demonstrated at 4 hours. This difference is promptly quenched since after 8 hours the migration rate of the two AMLs was similar.

SDF-1α treatment induced a statistically significant motility increase already after 2 hours treatment in cells from both AMLs. Significant difference persisted all along the experimental period (up until 8 hours). The effects induced by SDF-1α were abolished by the SDF-1α-receptor antagonist plerixafor, while no significant effects were induced by plerixafor alone.

**Fig 6:**
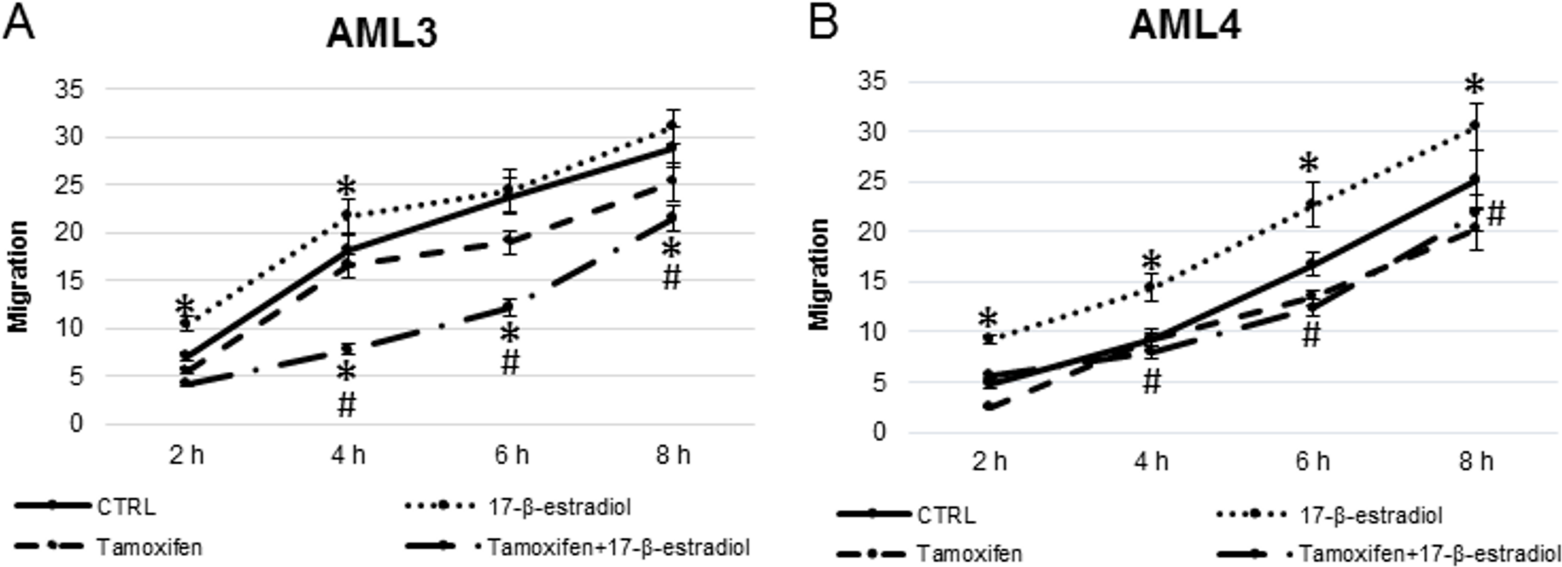
Wound healing assay in presence of 17-β-estradiol 1 nM and/or tamoxifen 2 μM. Data are expressed as the percentage of migration *vs* t = 0 h. * = P<0.05 vs control; ^#^ = P<0.05 vs 17-β-estradiol.

Two-dimensional migration was significantly modulated by 17-β-estradiol (Fig 6), although some differences in migration patterns were evident. In fact, AML3 (A) of male origin, showed a significant motility increase in the first 4 hours of incubation with 17-β-estradiol, and a prevalent logarithmic pattern. AML4 cells, of female origin (B), showed an exponential pattern and a significant increase in estradiol induced migration respect to the untreated control, evident at any of the time point considered. The treatments with the ER-antagonist tamoxifen had no influence on two-dimensional motility of both cell types, while it was able to abolish the effects of estradiol.

### Three-dimensional migration assays

**Fig 7:**
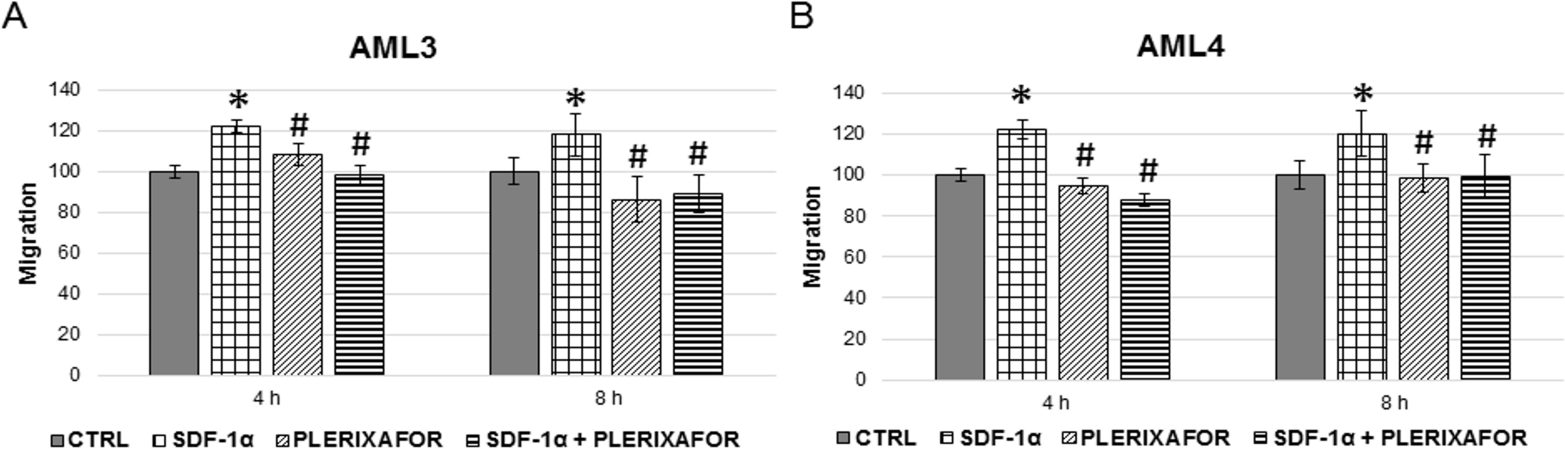
Three-dimensional migration (SDF-1α). Cells were incubated with SDF-1α (100 ng/mL) and its receptor blocker plerixafor (100 nM) or a combination of both for 4 or 8 hours. In each experimental condition cells were counted in 5 fields *per* insert. Data are expressed as the percentage of migration *vs* control. * = P<0.05 vs control; ^#^ = P<0.05 vs SDF-1α.

Cells were seeded in the upper chamber of a transwell filter and allowed to migrate for 4 or 8 hours under either basal conditions, or in response to stimuli added in the lower chamber. Fig 7, panel A and B, shows that the number of migrating cells significantly increased in response to SDF-1α.

Data were similar for cells originated from the two AMLs and were significant after 4 hours of continuous exposure. SDF-1α stimulation was completely abolished by the SDF-1α-receptor antagonist plerixafor, while no differences were shown for longer exposures.

**Fig 8:**
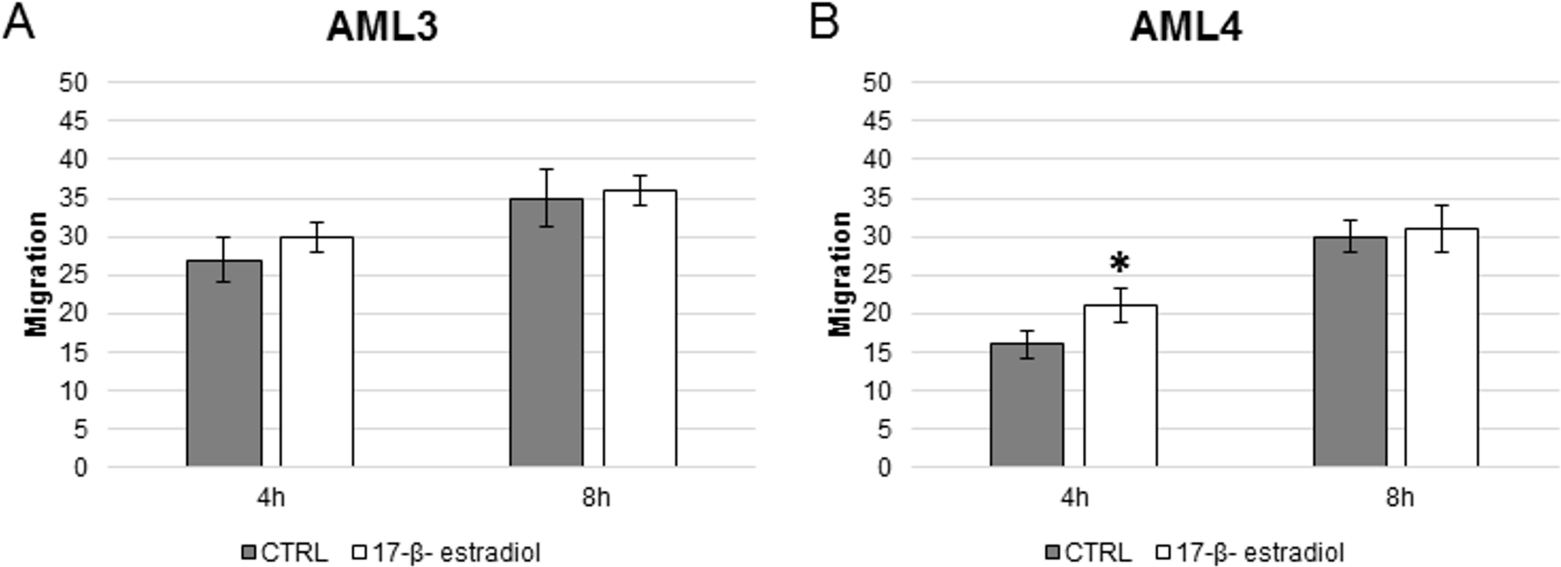
Three-dimensional migration (17-β-estradiol). Cells were incubated with 17-β-estradiol (1 nM) for 4 and 8 hours. Migration is expressed in arbitrary units. * = P<0.05 vs control.

The transwell migration experiments performed in presence of 17-β-estradiol indicated a different behavior between cells originating from the two AML. AML3 cells of male origin needed at least 8 hours of stimulation before a significant difference in migration could be appreciated (Fig 8A). AML4 cells (Fig 8B) responded to the stimulation at 4 hours, while after 8 hours no differences respect to the control were evident.

### Metalloproteases activity in supernatant derived from 3D-migration test

**Fig 9:**
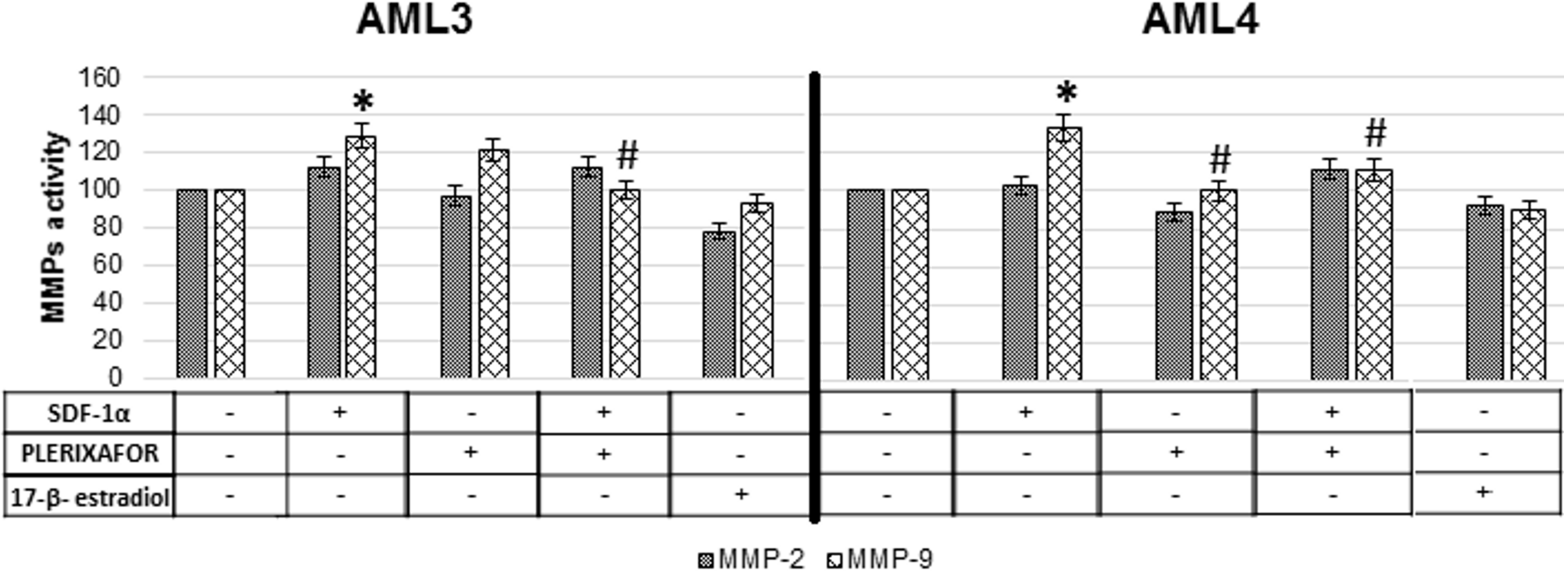
Metalloproteases activity in supernatant collected from 3D-migration test. Data are expressed as the relative activity calculated by densitometric analyses. * = P<0.05 vs control; ^#^ = P<0.05 vs SDF-1α.

The activity of two metalloproteases involved in malignant phenotype acquisition, MMP-2 and MMP-9, was measured by zymography in the supernatant collected at the end of the 3D-migration test (4 hours) (Fig 9).

While the diverse treatments induced no significant difference in MMP-2 activity, SDF-1α enhanced MMP-9 enzymatic activity. This data was in accordance with the increased migration induced by SDF-1α. Coherently, plerixafor inhibited MMP-9 activity increase provoked by SDF-1α. No difference was induced by incubation with 17-β-estradiol.

**Fig 10:**
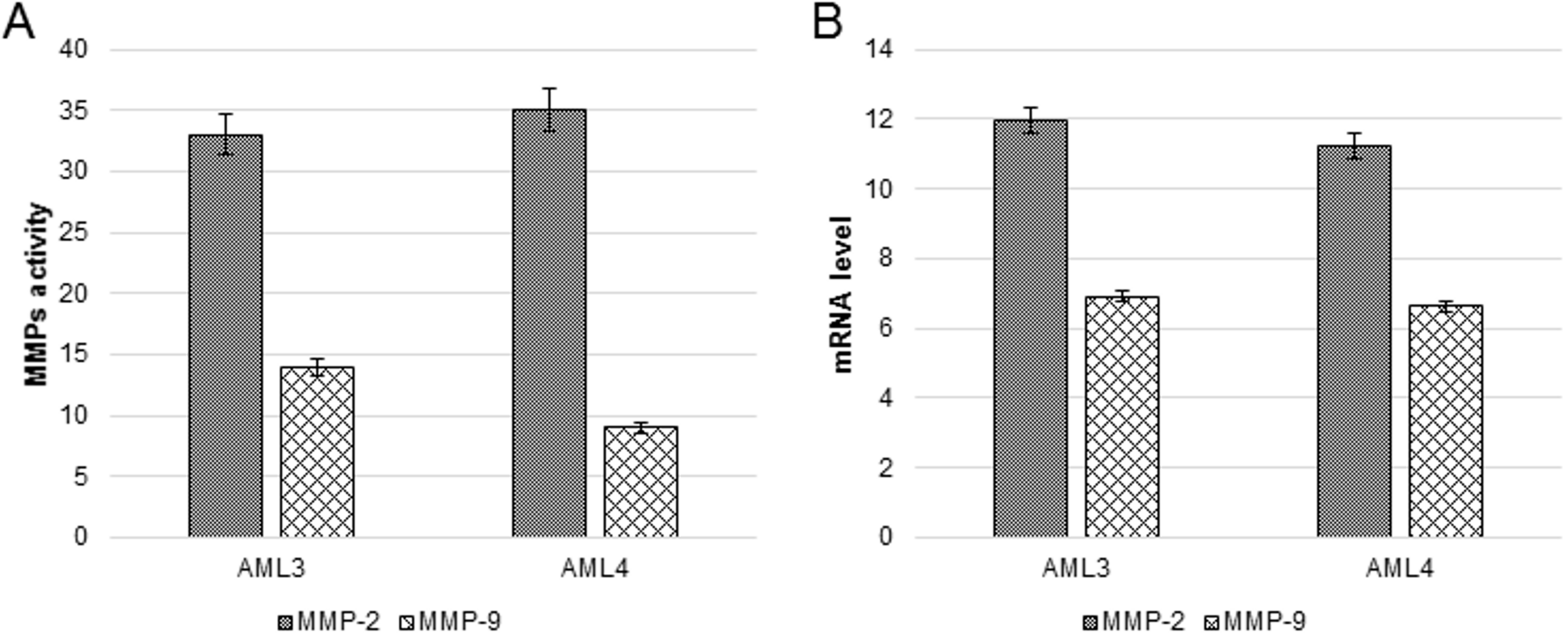
Absolute MMP-2 and MMP-9 activity (A) and their corresponding mRNA expression (B) in basal conditions. Data are expressed as the activity calculated by densitometric analyses. The gene expression is shown as the level of expression normalized by the housekeeping 18S rRNA. The difference in MMP-2 *vs* MMP-9 activity or expression is always significant (P<0.001).

Fig 10, panel A, shows absolute MMPs activity (not normalized) and demonstrates that MMP-2 activity, in basal conditions, is higher than MMP-9 activity for both AMLs. Consistently, Fig 10B, shows coherent mRNA expression for both enzymes.

## Discussion

In this paper we describe for the first time the behaviour of primary AML cells, originating from male and female patients, in terms of proliferation and migration *in vitro*, both in basal conditions and in response to environmental stimuli. Our findings clearly demonstrate that primary AML cells are able to migrate *in vitro*.

The main limit of this study was, by far, the small sample number. It is worth underlying, though, that AML is a rare disease and it is quite difficult to come across AML of male origin, and to isolate and grow primary cells, what we actually did. The cellular composition of the cultures were similar, as demonstrated by immunofluorescence and shown in Fig 1. Overall, this phenotype confirmed the mixed (either muscle, epithelioid, lipomatous and mesenchimal) nature of the cultures, respectively, according to the heterogeneous nature of AML [24]. None of the cell type seemed to be predominant; we consider the miscellaneousness of the two cultures an advantage in a pharmacological study, since the complexity of the tumor should be taken into account. Deeming the characterization of the cells of outstanding interest, we conducted also an exhaustive investigation that considered the TSC mutational status, evidencing no deletion in TSC2 in any of the two samples.

The original aim of this study was to understand if AMLs, independently from their TSC mutational status, were able to migrate and if there was the possibility to modulate this process pharmacologically. In particular, we focused on estrogen because of the underlying hypothesis that in females during childbearing age AML cells might migrate to the lungs and other organs. We also investigated SDF-1α since this factor is usually associated to migration and homing of several cell types [31,21]. We have shown that both AML3 (male origin) and AML4 (female origin) have analogous significant expression levels of estrogen and SDF-1α receptor mRNAs. Despite the presence of the estrogen receptors, we have observed that there was no estrogenic influence on proliferation of both AMLs. Therefore, the hormone therapy might not be optimal pharmacological choice to treat this pathology in female patients, and other options should be taken into consideration. Another aspect that we demonstrated in common for both AMLs was their migratory response to SDF-1α. In fact, we have shown its significant effect in increasing the bi- and three-dimensional migration of these cells. The specificity of this observation was confirmed by the effect of the treatment with the selective SDF-1α receptor antagonist plerixafor, which completely abolished SDF-1α migratory stimulation.

Our results are in accordance with the well known role of SDF-1α in cell migration, and might support the hypothesis that the host microenvironment tissue stimuli could exert a specific chemotactic signal to promote homing of AML cells [32].

Although some common properties between the two AMLs have been described so far, we report that other aspects seem to differentiate the cells originating from male from those deriving from female patients. We showed that the basal unstimulated migration of male AML3 had a more rapid onset, respect to female AML4. This difference in spreading was striking already 4 hours after seeding, and was particularly evident in the ability to invade, as demonstrated by three-dimensional assays. Alongside with this observation, we also report that the higher basal migration of AML3 is less influenced by estrogen, while spreading of AML4 cells is significantly increased already after 2 hours incubation with estradiol 1 nM.

In order to clarify the short-term effects of estrogen, we investigated the expression of ERα, ERβ and GPR30. It is worth to underline an abundant mRNA expression of GPR30 [33], which might likely mediate the early response to estradiol that we observed.

In this work we present some data on the response of AML cells to hormonal stimuli. While many Authors have thoroughly described the long term effects of these stimuli [34, 35], their influence on the colonization potential and short term effects needs further research. In particular, our attention focused on GPR30 role in these processes, and we have chosen the timing in order to better analyze the responses referred to this receptor pathway. The multiple effects of estrogens can be explained by different modulation of transcription and by rapid signaling events that are not associated with altered gene transcription [34]. GPR30 hormone stimulation is able to induce rapid MAPK activation pathway and ERK1/2 phosphorylation via MMP-EGFR and is responsible for several cell responses and signaling.

We have included to this paper some Western blot experiments that demonstrate that E2 stimulation is able to increase ERK1/2 phosphorylation in AML4. Noteworthy, both AML3 and AML4 constitutively express significant levels of ERK1/2 and pERK. In particular, AML3 has a basal level of pERK higher than AML4, and this might explain the reduced effects of E2 on pERK/ERK observed in AML3. These data are in accordance with the 2D and 3D migration data shown in Fig 6 and Fig 8 and justify why the early response to estradiol in migration of AML4 cells is not related to a higher mRNA expression compared to AML3.

ERK protein is activated when tuberin function is lost [36], and estrogen-mediated non-genomic ERK signaling activated by GPER is involved in cell viability and motility of TNBC cells [37].

An interesting aspect of GPR30-E2 stimulation is the activation of the PI3K-Akt signaling pathway via mTOR and S6K that leads to different activity on DNA transcription and on proliferation. This pathway is activated from both E2 and tamoxifen, and it could explain, at least in part, the data shown in Fig 6.

Analysis of the culture media collected from three-dimensional migration experiments revealed that the stimulation of migration induced by SDF-1α was accompanied by an augmented release of MMPs. These effects were consistently reverted by the selective receptor antagonist plerixafor. Strikingly, such an increase in MMPs production was not observed in estradiol-stimulated conditions, despite a significant increase in cell migration. We therefore speculate that, while SDF-1α -induced migration occurs through metalloproteases production, the migratory response to estrogen most likely does not involve MMPs activity.

Noteworthy we have shown that tamoxifen induced unexpected effects on migration of the two cell types. The peculiar mechanism of action of this drug, which can bind both intracellular and membrane estrogen receptors, might give us some hints in the interpretation of our results.

Our work demonstrates that primary AML cells migrate and produce active metalloproteases, both aspects being consistent with the theory claiming these cells can migrate and invade other tissues, and for some yet unknown reasons colonize the lung.
